# Involvement of FAK-ERK2 signaling pathway in CKAP2-induced proliferation and motility in cervical carcinoma cell lines

**DOI:** 10.1038/s41598-017-01832-y

**Published:** 2017-05-18

**Authors:** Qi-sang Guo, Yu Song, Ke-qin Hua, Shu-jun Gao

**Affiliations:** 1 Cervical Diseases Diagnosis & Treatment Center, Obstetrics and Gynecology Hospital, Fudan University, Shanghai, 200011 P. R. China; 2 Department of Gynecology, Obstetrics and Gynecology Hospital, Fudan University, Shanghai, 200011 P. R. China; 3 Shanghai Key Laboratory of Female Reproductive Endocrine-Related Disease, Fudan University, Shanghai, 200011 P. R. China

## Abstract

Cervical carcinoma is the fourth most common cause of death in woman, caused by human papillomavirus (HPV) infections and arising from the cervix. Cytoskeleton-associated protein 2 (CKAP2), also known as tumor-associated microtubule-associated protein, has been linked to tumorigenic effects. In the present study, we screened CKAP2 as a new candidate gene which promotes development of cervical carcinoma, in two independent datasets (TCGA and GSE27678). Results showed that CKAP2 expression was significantly up-regulated in cervical cancerous tissues compared with normal counterparts. Gene set enrichment analysis (GSEA) showed that metastasis, cell cycle and FAK pathways were related with elevated CKAP2 expression. Knockdown of CKAP2 expression significantly inhibited cell proliferation, migration and invasion both in HeLa and C-33A cells. And depletion of CKAP2 down-regulated the expression of metastasis and cell cycle related proteins as well as the phosphorylation of ERK2 (p-ERK2), except E-cadherin. *In vivo* experiment revealed that knockdown of CKAP2 inhibited C-33A cells proliferation. However, FAK inhibitor PF-562271 and ERK2 inhibitor VX-11e treatment significantly inhibited CKAP2 overexpression-induced cell proliferation, migration and invasion in SiHa cells. In conclusion, our study suggests that CKAP2 acts as a functional oncogene in cervical carcinoma development and may exert its function by targeting FAK-ERK2 signaling pathway.

## Introduction

Cervical carcinoma is the fourth most prevalent female malignant disease that affects women of different ages and backgrounds worldwide. There are more than 500,000 new cases diagnosed and approximately 275,000 deaths due to cervical cancer each year^1^. The most important risk factor for cervical carcinoma is persistent human papilloma virus (HPV) infection^2^, especially for cervical squamous cell carcinoma, which accounts for approximate 80% of cervical carcinoma^3^. The 5-year survival rates for advanced stage patient remains at less than 30% because of metastatic spread of cancer cells to distant area such as pelvic lymph node^2, 4^. Recent molecularly targeted therapeutics have shown potential in decreasing metastasis and improving survival for several human malignancies^5, 6^. Therefore, an increased understanding of the molecular targets and pathways of cervical carcinoma progression and metastasis is necessary.

The gene for cytoskeleton-associated protein 2 (CKAP2), also known as tumor-associated microtubule-associated protein, expresses cell cycle dependently at the late G1/S phase and reaches the peak time during the G2/M phase^7^ and plays important functions in cell proliferation, particularly during mitosis^8, 9^. It has been found up-regulated in malignancies, including human gastric adenocarcinomas^10^, diffuse large B-cell lymphomas^11^, hepatocellular carcinoma^12^ and breast cancer^13^. CKAP2 enhances wild-type p53 activity and triggers G1 arrest and apoptosis in a p53-dependent manner^14^. CKAP2 was identified in the previous study as a molecule that was significantly associated with worse relapse-free survival in early-stage breast cancer^13^. Although CKAP2 was reported to be up-regulated in malignancies, the exact biologic functions of CKAP2 in cervical carcinoma have not been fully identified.

Focal adhesion kinase (FAK) is a non-receptor tyrosine kinase that plays an important role in signal transduction pathways that are initiated at sites of integrin-mediated cell adhesions and by growth factor receptors. Although FAK expression is low in benign proliferative lesions, FAK overexpression occurs in some human malignant tumors, including squamous cell carcinoma of the larynx^15^, invasive squamous cell carcinoma^16^ and malignant melanoma^17^. Several studies have shown that FAK functions as part of a cytoskeleton-associated network of signaling proteins, which act in combination to transduct integrin-generated signals to the ERK/JNK mitogen-activated protein (MAP) kinase cascades, and promotes epithelial proliferation^6, 18, 19^. In addition to survival and proliferation, FAK signaling is linked to spreading and migration processes. Inhibition of FAK results in the prevention of Src-mediated ERK2 and JNK activation and a reduction in MMP-2, indicating a role for Src-FAK cooperation in invasion^18^. FAK overexpression is not restricted to invasive phenotype, but rather appears to be a marker for malignant transformation in breast and cervical carcinomas^16^.

In the current study, we showed that the expression level of CKAP2 was higher in cervical carcinomas tissues than in adjacent tissues. We also showed that knockdown of CKAP2 inhibited the proliferation, migration and invasion of cervical carcinomas cells. The involved possible mechanism was also explored. Taken together, these results suggest that CKAP2 could regulate cervical carcinogenesis and may serve as a potential target for cervical carcinomas therapies.

## Materials and Methods

### Tissue samples

A total of 247 patients enrolled in this study underwent resection of the primary cervical carcinoma at Obstetrics and Gynecology Hospital, Fudan University (Shanghai, China). The tumor stage was classified by two experienced gynecological oncologists according to the International Federation of Gynecology and Obstetrics (FIGO) staging system for cervical cancer. Clinical and pathological variables analyzed are shown in Table 1. The study was approved by Research Ethics Committee of Obstetrics and Gynecology Hospital, Fudan University and written informed consent was obtained from all patients. Tumor samples and according normal tissues were immediately frozen in liquid nitrogen and kept at −80 °C until used. All experiments were performed in accordance with the guidelines and regulations of Research Ethics Committee of Obstetrics and Gynecology Hospital, Fudan University.

**Table 1:** Relationship between CKAP2 and clinical characteristics of cervical carcinoma patients.

Factor	Characteristic	CKAP2 expression (N)		*P*-values
		Lower	Higher	
Age (years)	<35	60	42	0.023
	>=35	62	83	
Histology	Squamous	105	110	0.653
	Adenocarcinoma	17	15	
HPV	HPV16	78	80	0.536
	HPV18	27	31	
	Others	17	14	
FIGO stage	Ib~IIa	21	6	0.005
	IIb~IIIa	101	119	
Histological differentiation	Well	5	8	0.521
	Moderate	78	73	
	poor	39	44	
Lymph node metastasis	Negative	115	78	<0.0001
	Positive	7	47	
Recurrence	Yes	27	59	0.002
	No	95	66	
Tumor maximum diameter (cm)	<4	98	80	0.006
	>=4	24	45	

Pearson Chi-square test.

### Cell culture

Five cervical carcinoma cell lines (C-33A, CaSki, HeLa, SiHa and C4-1) obtained from the Cell Bank of Academia Sinica (Shanghai, China) were used in this study. All the cells were cultured in Dulbecco’s modified Eagle’s medium (DMEM, Hyclone Company, USA), in which 100 U/mL penicillin, 100 μg/mL streptomycin and 10% heat-inactivated FBS (Gibco BRL) were supplemented. All the incubator (Thermo Fisher Scientific Inc.) was set to 37 °C, 100% humidity and 5% CO_2_. The cell lines used in our study were validated by STR and showed no Cross contamination.

### Construction of stable transfection

To knockdown the expression of CKAP2 in selected cell lines, shRNAs targeting three human CKAP2 mRNA positions were synthesized, including shRNA-1 (position 1, GTTCTATCTTGGCGCTAAA), shRNA-2 (position 2, GCCTGCTTCATTGTCTAAT) and shRNA-3 (position 3, GCAGTTTGATGGAACAAAT). The shRNA sequences were cloned into the pLKO.1-EGFP lentiviral vector. A non-specific scramble shRNA was cloned into the pLKO.1-EGFP lentiviral vector served as negative control (shNC). To overexpress the expression of CKAP2 in selected cell lines, CKAP2 coding sequence was cloned into the pLVX-Puro lentiviral vector. A blank pLKO.1-EGFP lentiviral vector was used as negative control (NC). The constructs were then co-transfected into HEK 293T cells with lentiviral packaging vectors by using lipofectamine 2000 (Invitrogen Life Technologies, Gaithersburg, MD, USA) according to the manufacture’s instruction. The recombinant lentivirus vector was collected 48 h after transfection and used to infect HeLa, C-33A and SiHa cells. After 48 h of infection, cells were used for assays as follows.

### CCK-8 assay

The effects of CKAP2 on cervical carcinoma proliferation were measured by a Cell Counting Kit-8 (CCK-8) assay kit. Briefly, HeLa and C-33A were plated at a density of 1 × 10^4^ cells in 96-well plastic plates and further incubated for 24 h, 48 h and 72 h. CCK-8 solution was added on indicated time points to each well and incubated for 1 h. The cell viability in each well was determined by reading the absorbance of the culture medium at a test wavelength of 450 nm and a reference wavelength of 630 nm (Thermo Scientific, USA).

### *In vitro* cell migration and invasion assay

Migration assay was performed using Transwell chamber (Greiner Bio-One, Frickenhausen, Germany) as described in the manufacturer’s protocol. Briefly, cells were trypsinized, washed, and kept suspended in DMEM. Serum-free DMEM with 5 × 10^4^ cells/well was filled in the upper wells of the chambers and DMEM with 10% FBS was added to the lower wells of the chambers. Then, the chamber was placed in 37 °C incubator for 24 h. After 48 h incubation, cells on the upper well were wiped off by the Q-tip. The cells attached to the lower surface were washed with PBS, fixed in 4% paraformaldehyde and stained with 0.5% methylrosanilnium chloride solution for 30 min. Invasion assay was performed using Transwell chamber (Greiner Bio-One, Frickenhausen, Germany) coated with Matrigel (BD, San Diego, CA, USA) as described above. The cells attached to the lower surface were washed with PBS, fixed in 4% paraformaldehyde and stained by 0.5% crystal violet. Images of the cells were captured and cell numbers were counted under a microscope (Olympus Corporation) with magnification of ×400.

### Reverse transcription-quantitative polymerase chain reaction

Total RNA was extracted from cervical carcinoma tissues and cultured cells after different treatments with TaKaRa MiniBEST Universal RNA Extraction Kit according to the manufacturer’s instructions (TaKaRa, Japan) and contaminating DNA was depleted with RNase-free DNase (Qiagen, Valencia, CA). Total RNA (1 µg) was reverse transcribed into cDNA with PrimeScript™ II 1st Strand cDNA Synthesis Kit (TaKaRa, Japan) and an aliquot of cDNA mixture (0.2%) was used as polymerase chain reaction (PCR) templates. Primers were designed and purchase from Applied Biosystems and list in Table 2. Real-time PCR was performed on ABI 7500 (Applied Biosystem, Foster City, CA, USA) thermal cycler using a standard SYBR Green PCR kit (Thermo Fisher Scientific). Specificity of amplification products was confirmed by melting curve analysis (Supplementary Figs 1–4). Relative quantification of the gene expression was performed by normalization of the signals of different genes with the GAPDH signal.

**Table 2:** Primes sequences used in this study.

Gene	Sequences
CKAP2-forward	5′-GCCCAAAGAAACCTCGGAAG-3′
CKAP2-reverse	5′-GCAGGCTCATGCTGAGTAAC-3′
PCNA-forward	5′-GGTGTTGGAGGCACTCAAGG-3′
PCNA-reverse	5′-CAGGGTGAGCTGCACCAAAG-3′
MMP-2-forward	5′-TTGACGGTAAGGACGGACTC-3′
MMP-2-reverse	5′-GGCGTTCCCATACTTCACAC-3′
MMP-9-forward	5′-AAGGGCGTCGTGGTTCCAACTC-3′
MMP-9-reverse	5′-AGCATTGCCGTCCTGGGTGTAG-3′
Snail -forward	5′-TTCCTGAGCTGGCCTGTCTG-3′
Snail-reverse	5′-TGGCCTGAGGGTTCCTTGTG-3′
E-cadherin-forward	5′-GAGAACGCATTGCCACATACAC-3′
E-cadherin-reverse	5′-AAGAGCACCTTCCATGACAGAC-3′
GAPDH-forward	5′-CACCCACTCCTCCACCTTTG-3′
GAPDH-reverse	5′-CCACCACCCTGTTGCTGTAG-3′

### HPV detection and typing

Cervical carcinoma tissues were further tested for HPV using the SPF_10_-DNA enzyme immunoassay (DEIA)/reverse hybridisation line probe assay (LiPA_25_)-polymerase chain reaction (PCR) system (version 1: produced at Laboratory Biomedical Products, Rijswijk, The Netherlands), which detected 14 HR (16/18/31/33/35/39/45/51/52/56/58/59/66/68/73) and 11 low-risk (LR) HPV types (6/11/34/40/42/43/44/53/54/70/74). Specimens that were HPV-DNA positive by DEIA but did not yield an HPV type by the LiPA_25_ were further analyzed by sequencing as previously described^20^. When no type could be assigned even after performing sequencing, HPV was labeled as HPV undetermined.

### Western blotting

Cultured cells after different treatments were homogenized with lysis buffer (Beyotime Biotechnology, Jiangsu, China) and kept on ice for 25 min. Proteins (15 µg) after measured by BCA Protein Assay Kit (Beyotime Biotechnology, Jiangsu, China) were separated by 10% SDS-PAGE and electroblotted to polyvinylidene difluoride membranes membranes (Roche, Basel). The membranes were blocked with 5% fat-free dry milk in TBST (TBS, 0.1% Tween 20) for 2 h at room temperature and subsequently incubated with primary antibody overnight at 4 °C with gentle agitation and horseradish peroxidase (HRP)-conjugated secondary antibody for 2 h at room temperature. All membranes were detected using enhanced chemiluminescence (Thermo Scientific).

### Immunohistochemistry

Tissue sections were initial treatment for deparaffinization and hydration and then heated in EDTA (pH 8.0) and antigen-retrieved in 10 mm citrate buffer for 5 min at 100 °C. The reaction of CKAP2 antibody (Abcam) was taken place 1 h at room temperature, following incubated by biotin-labeled secondary antibodies and slides were then developed using 3,3-diaminobenzidine (DAB; Shanghai Long Island, Co., LTD, China) solution and counterstained with hematoxylin staining (BASO, China). Immunohistochemical signals were calculated with the positive staining cells under a microscope (Olympus Corporation) with magnification of ×400.

### Tumor Xenografts

4-week-old male athymic nude mice (n = 12) were subcutaneously inoculated with 0.1 mL of C-33A cells (1 × 10^7^ cells/mL) infected with pLKO.1-EGFP-CKAP2 shRNA (n = 6) or pLKO.1-EGFP-shNC (n = 6) at one site of the right flank. Tumor growth was measured every three days for 27 days, the tumor volume was calculated according to the formula: tumor volume = (the shortest diameter)^2^ × (the longest diameter)/2. After that, the mice were killed and tumor tissues were weighed, excised, paraffin-embedded, formalin-fixed, and performed H&E staining and TUNEL. We confirmed that all methods were carried out in accordance with relevant guidelines and regulations and all experimental protocols were approved by Obstetrics and Gynecology Hospital, Fudan University.

### Bioinformatics analysis

RNA-Seq data from cervical carcinoma tissues and adjacent tissues was downloaded from The Cancer Genome Atlas (TCGA) and the NCBIs Gene Expression Omnibus (GEO, http://www.ncbi.nlm.nih.gov/geo/) are accessible through GEO Series accession number GSE27678 datasets following approval of this project by the consortium. 253 cervical carcinoma (median age 45, range from 20 to 88; 195 stage I-II and 58 stage III-IV; 76.7% of squamous cell carcinoma and 23.3% of adenocarcinoma) and 3 adjacent tissues of patients (median age 55, range from 40 to 55; 1 stage I and 2 stage II; 2 squamous cell carcinoma and 1 adenocarcinoma) were included in TCGA while 32 cervical carcinoma (21 high grade squamous intraepithelial lesions (HSIL) and 11 low grade squamous intraepithelial lesions (LSIL)) and 12 normal uterine cervix patients were included in GSE27678 datasets. To validate the correlation of CKAP2 and signaling pathway involved in pathogenesis in cervical carcinoma, a gene set enrichment analysis (GSEA) was performed to analyze the cervical carcinoma tumors in TCGA dataset. Firstly, GSEA generated an ordered list of all genes based on their association with CKAP2 expression. Then the predefined gene set (metastasis up, cell cycle and FAK pathways) received an enrichment score (ES) and nominal p-value. The expression level of CKAP2 was served as phenotype label, and “Metric for ranking genes” was set to Pearson Correlation.

### Statistical analysis

Values were presented as means ± SD. The SPSS 16.0 software system (SPSS, Chicago, IL, USA) was used for statistical analysis. All assays were performed in triplicate and repeated at least three times. The relationship between CKAP2 expression and clinicopathological characteristics was assessed using Pearson’s χ2 test. Survival curves were estimated by the Kaplan-Meier method. The log-rank test was used to estimate the statistical differences between survival curves. Cox proportional hazards analysis was performed to calculate the hazard ratio (HR) and the 95% confidence interval (CI) to evaluate the association between CKAP2 expression and survival. Multivariate survival analysis was carried out for all of the parameters that were significant in the univariate analysis using the Cox regression model. Statistical significance for the comparisons of the expression, migration, invasion and proliferation levels was determined using the Student’s t-test. P < 0.05 is considered statistically significant.

## Results

### CKAP2 expression is upregulated in human cervical carcinoma tissues

**Figure 1:**
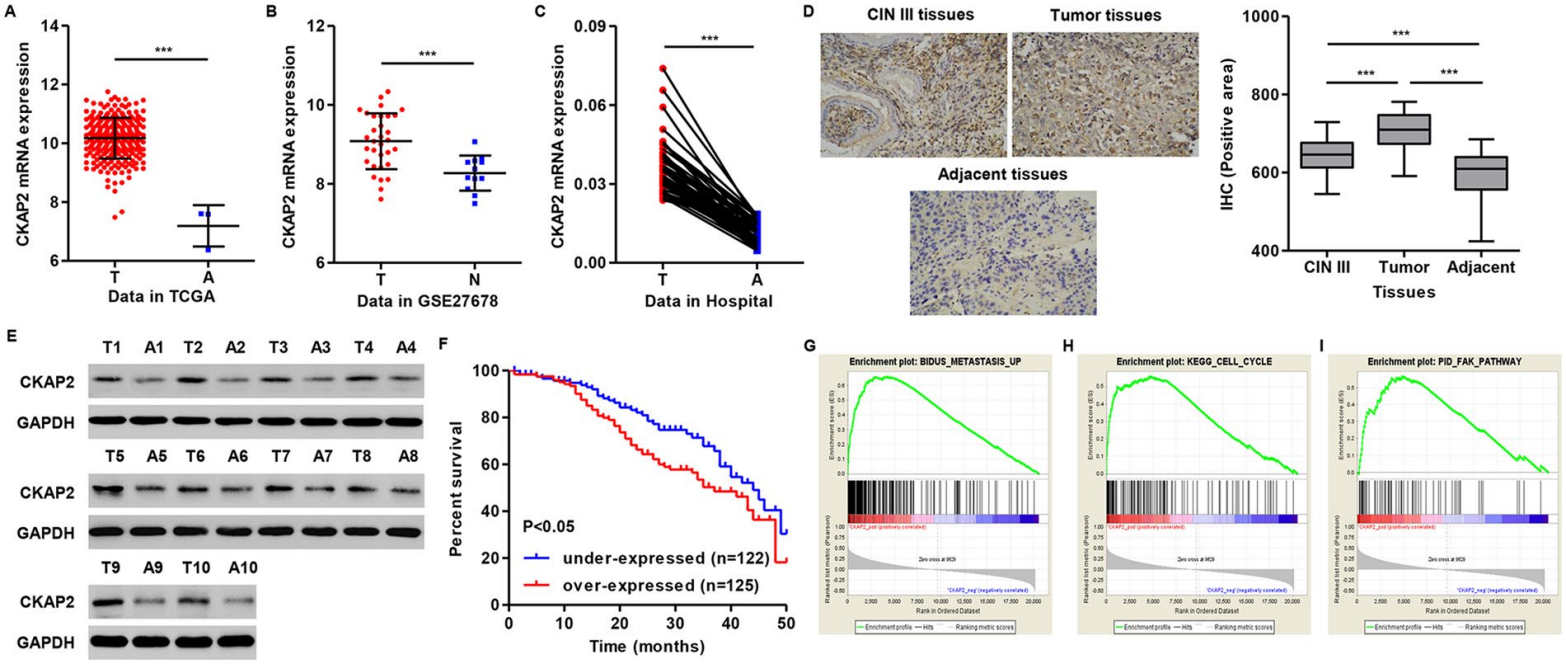
Expression of CKAP2 in cervical carcinoma tissues and cell lines. (**A**,**B**) Bioinformatics was also used to analyze the expression level of CKAP2 in TCGA data and GSE27678. (**C**) Comparison of expression level of CKAP2 between cervical carcinoma and adjacent tissues in Hospital by Real-time PCR. (**D**) Representative immunohistochemical staining for CKAP2 in cervical carcinoma, HPV-16-positive CIN III and adjacent tissues. (**E**) Expression of CKAP2 in ten primary cervical carcinoma tissues and their corresponding adjacent tissues measured by Western blot. (**F**) The survival time of cervical carcinoma specimens showed lower CKAP2 expression level patients were notably longer than that of higher CKAP2 patients. Genes in the metastasis up (**G**), cell cycle (**H**) and FAK pathway (**I**) showed significant enrichment in CKAP2-higher expression tissues versus CKAP2-lower expression tissues. T, tumor tissues; A, adjacent tissues; N, normal tissues; ***P < 0.001.

Bioinformatics analysis was used to measure CKAP2 expression in cervical carcinoma tissues and adjacent normal counterparts. The expression of CKAP2 was significantly upregulated in cervical carcinoma tissues when compared with the adjacent or normal counterparts in the TCGA data and GSE27678 database (Fig. 1A and B). Meanwhile, Real-time PCR also showed upregulated CKAP2 expression in cervical carcinoma tissues when compared with the adjacent tissues of patients in Obstetrics and Gynecology Hospital, Fudan University (Fig. 1C). To assess the protein levels of CKAP2 in human cervical carcinoma tissues, immunohistochemistry and Western blotting in 10 randomly selected cervical carcinoma (squamous cell carcinoma; FIGO stage, 5 Ib~IIa and 5 IIb~IIIa; HPV16) tissues were performed. High expression of CKAP2 was observed in human cervical carcinoma tissues compared with adjacent tissues (Fig. 1D and E). Moreover, we also analyzed the expression profile of CKAP2 in 10 HPV-16-positive CIN III tissues by immunohistochemistry analysis. The expression of CKAP2 in the CIN III tissues was generally increased compared with that in adjacent tissues and decreased compared with that in cervical carcinoma tissues (Fig. 1D).

Then we detected the clinical relevance of CKAP2 expression in cervical carcinoma, of the 247 human cervical carcinoma tissues were further classified into the high-CKAP2 group (n = 125) and low-CKAP2 group (n = 122) using the median expression value of CKAP2 as the cutoff point. The results showed that increased CKAP2 expression was significantly correlated with age, FIGO stage, lymph node metastasis, recurrence and tumor size, but not other clinical characteristics (Table 1). The survival time of cervical carcinoma patients showed that patients with under-expressed CKAP2 expression notably lived longer than patients with over-expressed CKAP2 expression (Fig. 1F). We next performed univariate and multivariate analysis of prognostic factors for overall survival with the Cox regression model (Table 3). We identified three prognostic factors, including FIGO stage, Lymph node metastasis and CKAP2 expression, can served as independent prognostic factors for poor overall survival.

**Table 3:** Univariate and multivariate analysis of clinicopathological factors for overall survival.

Clinicopathological	Subset	Univariate analysis			Multivariate analysis		
		HR	95% CI	p value	HR	95% CI	p value
Age (years)	<35 vs. >=35	1.116	0.628–2.121	0.589			
Histology	Squamous vs. Adenocarcinoma	1.128	0.477–2.691	0.748			
HPV	HPV16, HPV18 vs. Others	1.498	0.774–2.987	0.231			
FIGO stage	Ib~IIa vs. IIb~IIIa	3.618	1.935–6.984	<0.0001	2.318	1.157–4.646	0.018
Histological differentiation	Well vs. moderate, poor	0.725	0.400–1.382	0.472			
Lymph node metastasis	Positive vs. negative	4.253	2.424–8.308	<0.0001	2.236	1.144–4.926	0.005
Recurrence	Yes vs. No	1.708	0.802–3.813	0.139			
Tumor maximum diameter (cm)	<4 vs. >=4	1.374	0.624–2.491	0.539			
CKAP2 expression	High vs. Low	4.686	2.175–9.882	<0.0001	2.383	1.234–6.481	0.013

**Figure 2:**
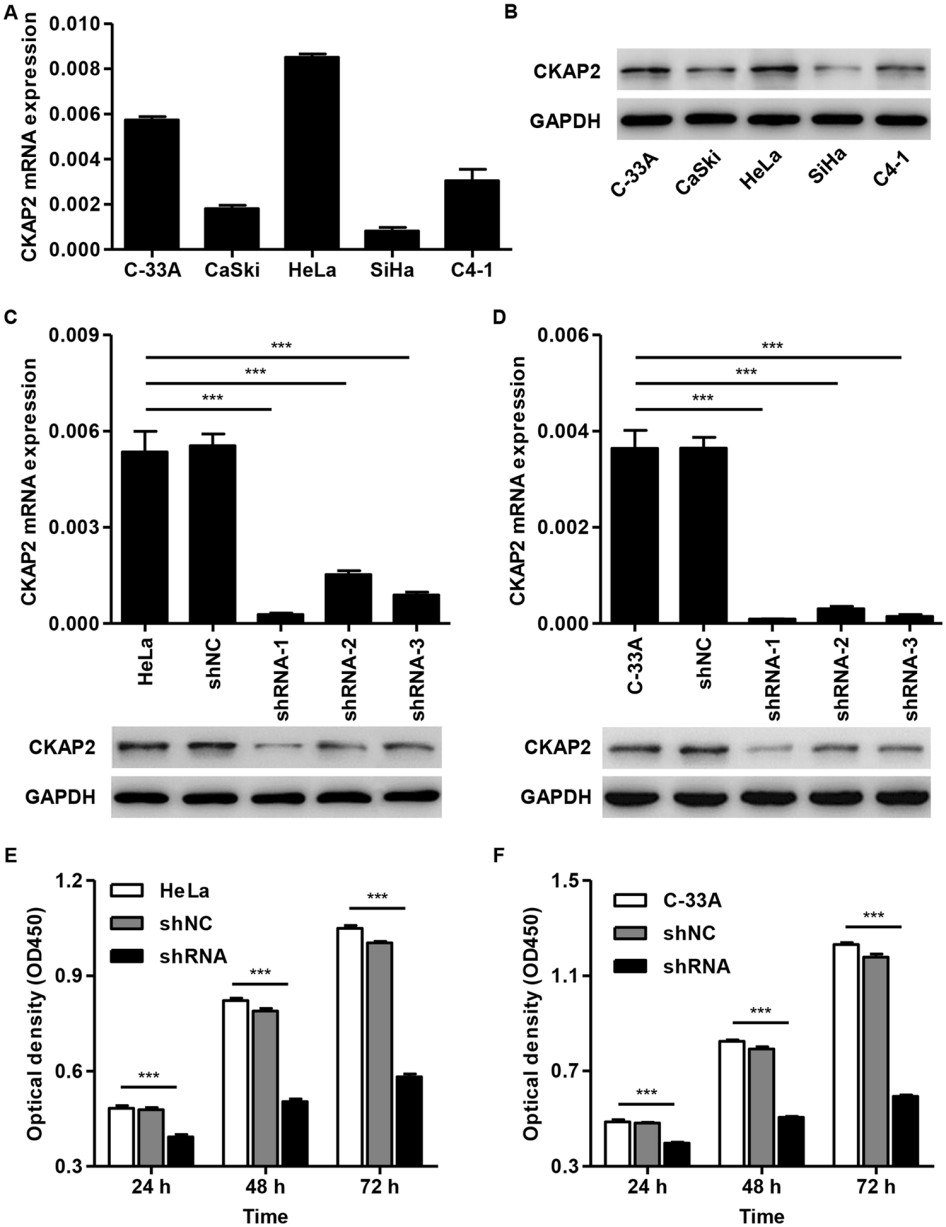
Effect of CKAP2 on cervical carcinoma cell proliferation. (**A**,**B**) The expression of CKAP2 in five human cervical carcinoma cell lines was assessed by Real-time PCR and Western blot. (**C**,**D**) Successful knockdown of CKAP2 in HeLa and C-33A cells was confirmed by Real-time PCR and Western blot at 48 h after infection with pLKO.1-EGFP-CKAP2 shRNA or pLKO.1-EGFP-shNC control lentivirus. (**E**,**F**) HeLa and C-33A cell proliferation was detected using CCK-8 assay at 24, 48, and 72 h. ***P < 0.001.

The exact pathways that CKAP2 may regulate in cervical carcinoma remain unclear. In order to probe the CKAP2-associated pathways in cervical carcinoma, we first performed GSEA using high throughput RNA-sequencing data of the cervical carcinoma tumors of TCGA database. Among all the predefined gene sets, the metastasis up, cell cycle and FAK signaling pathway was identified with the strongest association with CKAP2 expression (Fig. 2G–I).

### Effect of CKAP2 knockdown on cell proliferation *in vitro*

Next, we also detected the expression of CKAP2 in cervical carcinoma cell lines, including C-33A, CaSki, HeLa, SiHa and C4-1, in both mRNA and protein levels. As shown in Fig. 2A and B, HeLa and C-33A cells showed higher CKAP2 expression, and SiHa cells showed lowest expression of CKAP2 compared with other cervical carcinoma cell lines. These findings suggest that CKAP2 may associate with cervical carcinoma development and progression.

CKAP2 was silenced in HeLa and C-33A cells by infecting them with pLKO.1-EGFP-CKAP2 shRNA-1, -2, and -3, respectively. Real-time PCR and western blotting analysis of CKAP2 levels revealed that CKAP2 expression was decreased by 94.8 ± 1.2% and 77.7 ± 1.5% in HeLa cells and 97.5 ± 3.9% and 75.4 ± 1.2% in C-33A cells respectively following infection with pLKO.1-EGFP-CKAP2 shRNA-1 compared with control (Fig. 2C and D).

To assess the biological role of CKAP2 in cervical carcinoma cells, we investigated the effects of targeted knockdown of CKAP2 on cell proliferation. CCK-8 assay revealed that cell growth was significantly impaired in HeLa and C-33A cells infected with pLKO.1-EGFP-CKAP2 shRNA compared with controls (Fig. 2E and F). At 24, 48 and 72 h, the cell proliferation was significantly decreased by 18.6 ± 1.2%, 38.7 ± 1.4% and 44.5 ± 3.1% in HeLa cells and by 18.3 ± 0.9%, 38.7 ± 1.3% and 51.7 ± 2.8% in C-33A cells.

### CKAP2 inhibits tumorigenesis of cervical carcinoma cells *in vivo*

**Figure 3:**
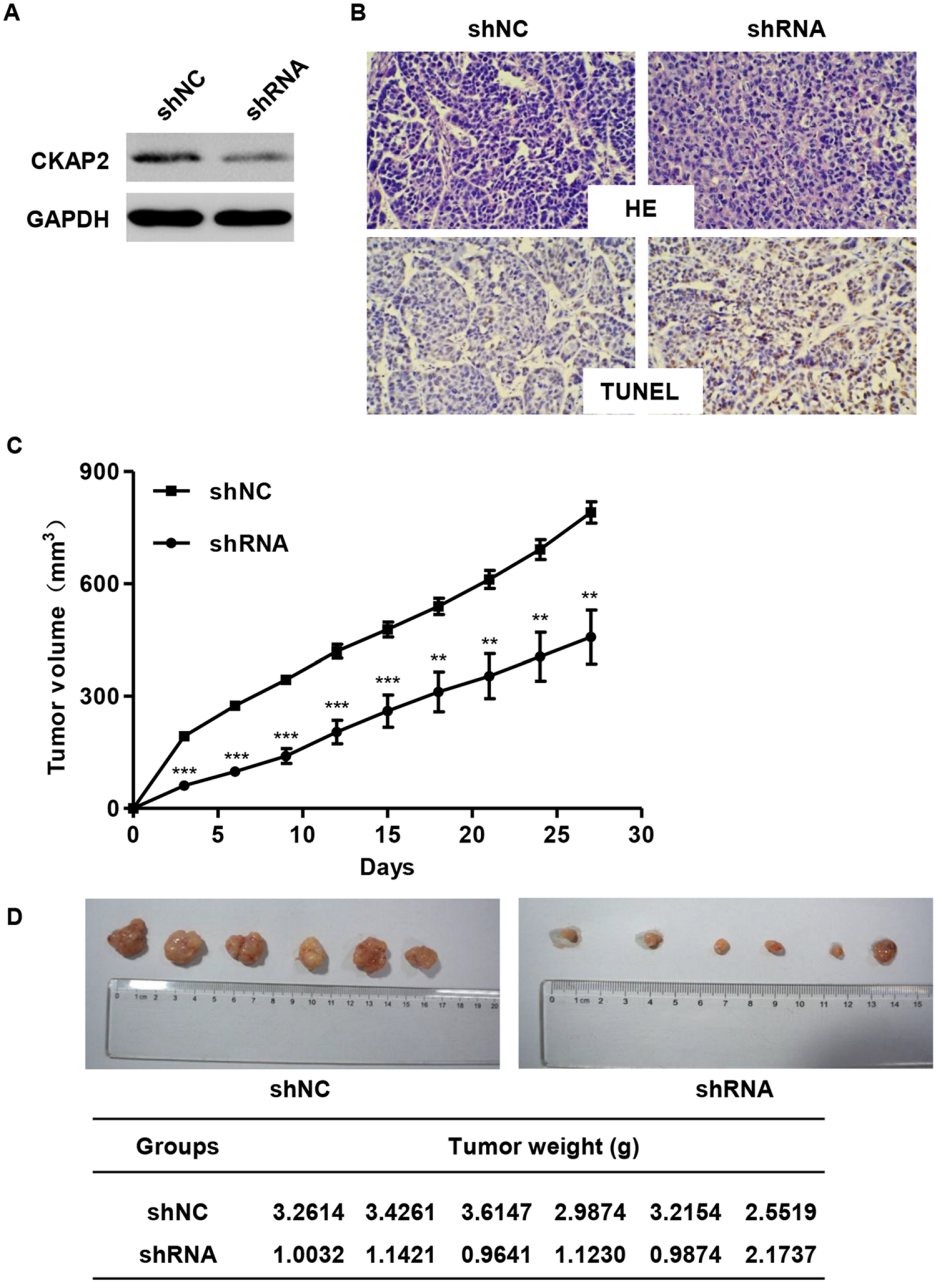
Effects of CKAP2 on tumor growth *in vivo*. (**A**) Expression of CKAP2 was measured by Western blot. (**B**) Tumors developed from pLKO.1-EGFP-CKAP2 shRNA infected C-33A cells showed higher TUNEL-positive cells than tumors developed by pLKO.1-EGFP-shNC cells. (**C**) The tumor volume was calculated every three days after injection of C-33A cells with pLKO.1-EGFP-CKAP2 shRNA or pLKO.1-EGFP-shNC. (**D**) Tumor weights are represented as means of tumor weights. **P < 0.01, ***P < 0.001.

To explore whether the level of CKAP2 expression affects tumorigenesis, pLKO.1-EGFP-CKAP2 shRNA and pLKO.1-EGFP-shNC stably-infected C-33A cells were inoculated into male nude mice. Western blotting was used to analyze CKAP2 protein expression in resected tumor tissues. CKAP2 levels in tumors formed from pLKO.1-EGFP-CKAP2 shRNA infected C-33A cells, exhibited decreased expression of CKAP2 than in tumors from control cells (pLKO.1-EGFP-shNC) (Fig. 3A). HE and TUNEL analysis was then performed in selected tumor tissues exhibited decreased apoptotic rate in tumors from pLKO.1-EGFP-CKAP2 shRNA infected C-33A cells than that from control cells (pLKO.1-EGFP-shNC) infected C-33A cells (Fig. 3B). 27 days after injection, the tumors formed in pLKO.1-EGFP-CKAP2 shRNA group were substantially smaller than those in the pLKO.1-EGFP-shNC group (Fig. 3C). Moreover, the mean tumor weight at the end of the experiment was markedly lower in the pLKO.1-EGFP-CKAP2 shRNA group (3.18 ± 0.37 g) compared to the pLKO.1-EGFP-shNC group (1.23 ± 0.47 g) (Fig. 3D). These results indicate that downregualtion of CKAP2 could inhibit tumor growth *in vivo*.

### Effect of CKAP2 knockdown on cell migration and invasion *in vitro*

**Figure 4:**
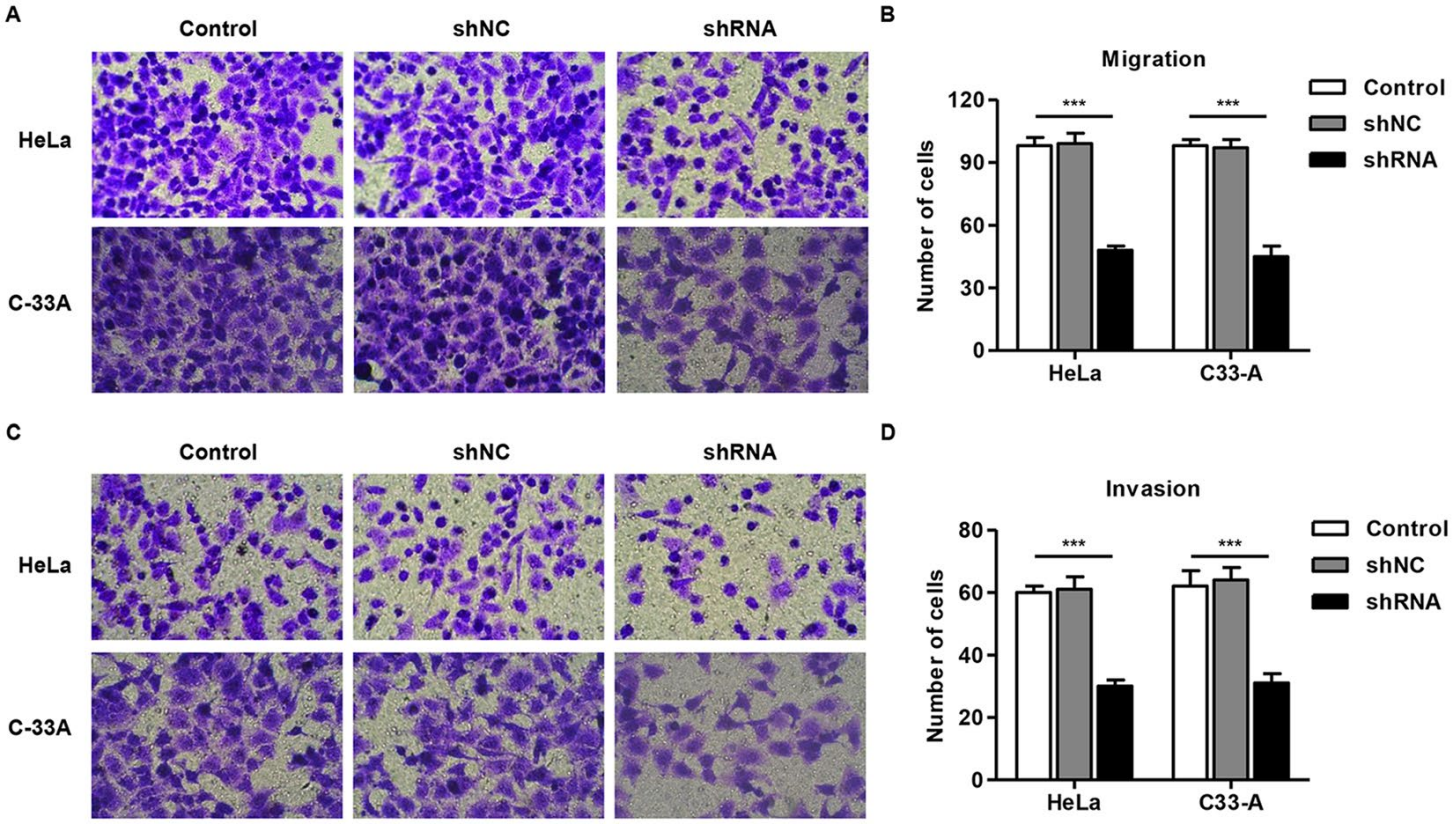
Effect of CKAP2 on cervical carcinoma cell migration and invasion. (**A**,**B**) The migration of HeLa and C-33A cells was performed by Transwell assay. (**C**,**D**) The invasion of HeLa and C-33A cells was performed by Transwell Matrigel assay. ***P < 0.001.

Cell invasion is a significant aspect of cancer progression, and involves the migration of tumor cells into contiguous tissues and the dissolution of extracellular matrix proteins. To investigate whether CKAP2 had a direct functional role in facilitating cell invasion in cervical carcinoma, we evaluated cancer cell migration and invasion through transwell assay after infection with pLKO.1-EGFP-CKAP2 shRNA. The number of migrated HeLa and C-33A cells infected with pLKO.1-EGFP-CKAP2 shRNA decreased by approximately 51 ± 3% and 54 ± 5% respectively in comparison with control cells (Fig. 4A and B). Meanwhile, the number of invaded HeLa and C-33A cells infected with pLKO.1-EGFP-CKAP2 shRNA both decreased by approximately 50% in comparison with control cells (Fig. 4C and D).

### Validation of GSEA analysis of TREM2 in cervical carcinoma cells

**Figure 5:**
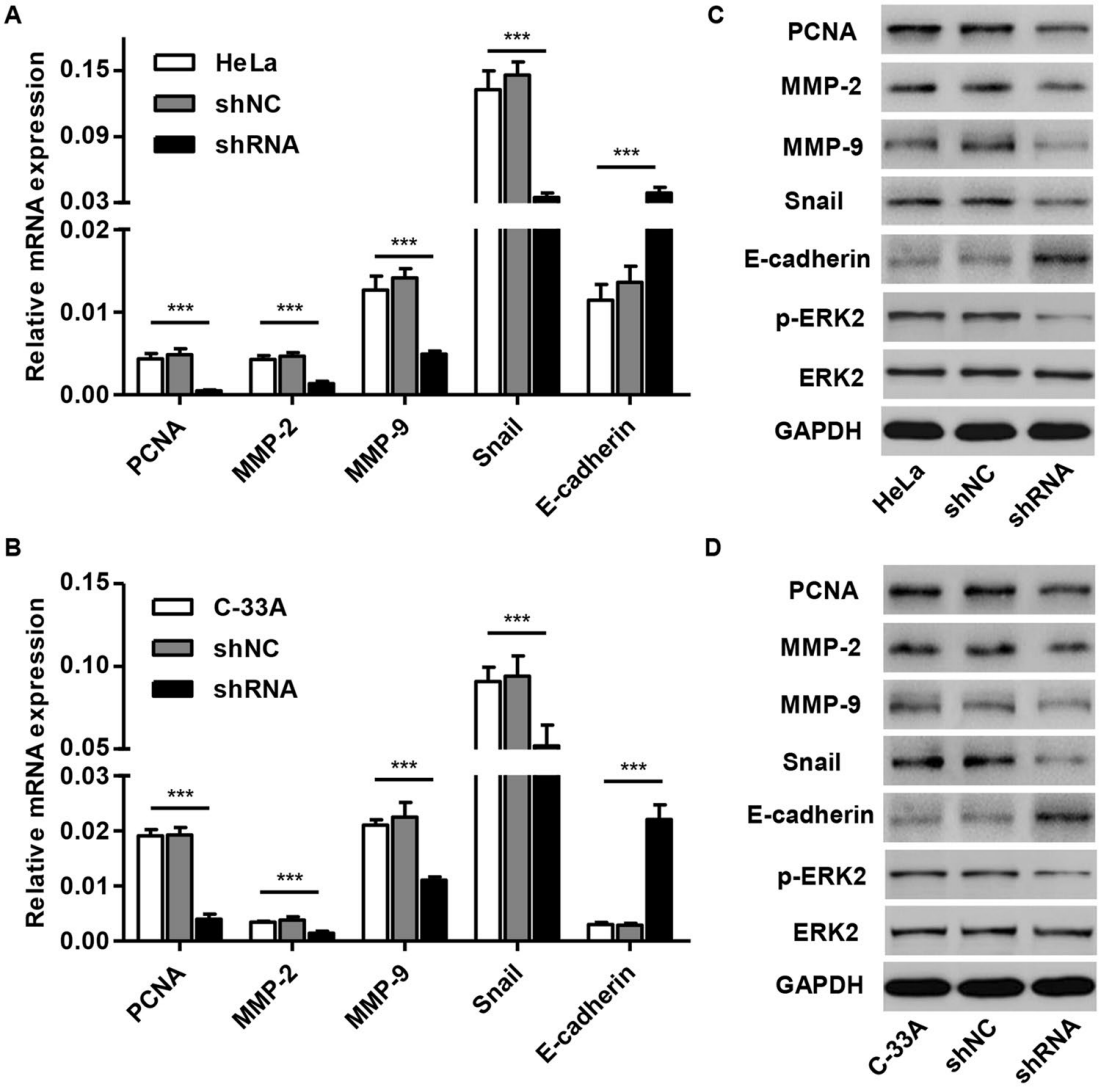
Mechanisms of CKAP2 exert their functions in cervical carcinoma cells. After treatment of HeLa and C-33A cells with pLKO.1-EGFP-CKAP2 shRNA, the expression of PCNA, MMP-2, MMP-9, Snail and E-cadherin was analyzed by Real-time PCR (**A**,**B**) and Western blot (**C**,**D**). ***P < 0.001.

To validate the results in GSEA, we performed Real-time PCR and Western blot analysis in cervical carcinoma cells. As shown in Fig. 5A and B, the mRNA expression of cell cycle related gene (PCNA) and metastasis related genes (MMP-2, MMP-9 and Snail) were significantly decreased in HeLa and C-33A cells infected with pLKO.1-EGFP-CKAP2 shRNA compared with controls as well as the expression of p-ERK2/ERK2. While E-cadherin mRNA level was increased in response to pLKO.1-EGFP-CKAP2 shRNA infection. The similar results were also found in our Western blot analysis in both HeLa and C-33A cells (Fig. 5C and D).

### Effect of PF-562271 and VX-11e on CKAP2 overexpression-induced migration and invasion *in vitro*

**Figure 6:**
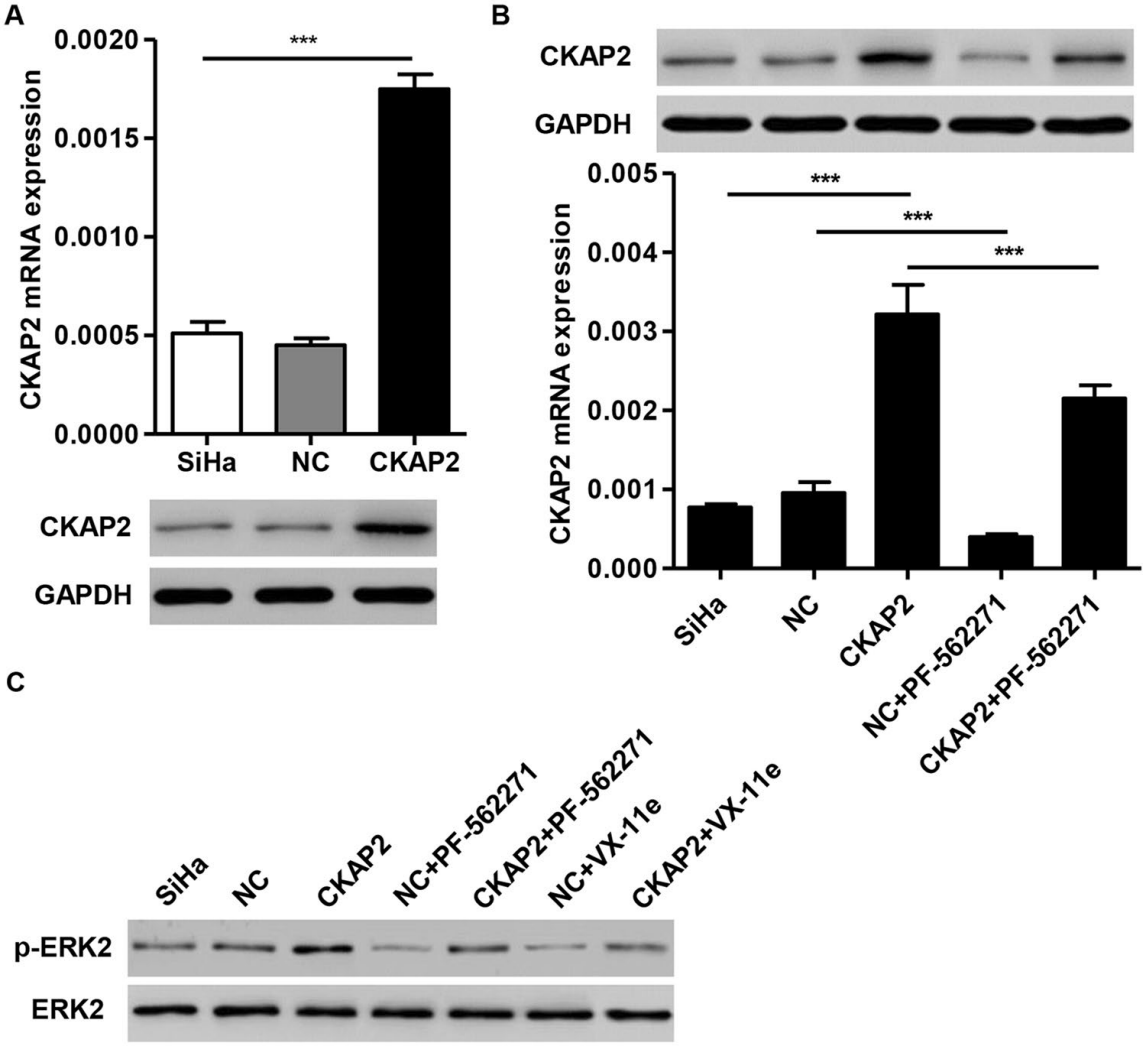
Effects of PF-562271 or VX-11e treatment on CKAP2 and p-ERK2 expression. (**A**) Successful overexpression of CKAP2 in SiHa cells was confirmed by Real-time PCR and Western blot at 48 h after infection with pLVX-Puro-CKAP2 or empty vector. (**B**) SiHa cells were treated with 10 μM of PF-562271, and the expression of CKAP2 was measured by Real-time PCR and Western blot. (**C**) SiHa cells were treated with 10 μM of PF-562271 or VX-11e, and the expression of p-ERK2 was measured by Western blot. ***P < 0.001.

**Figure 7:**
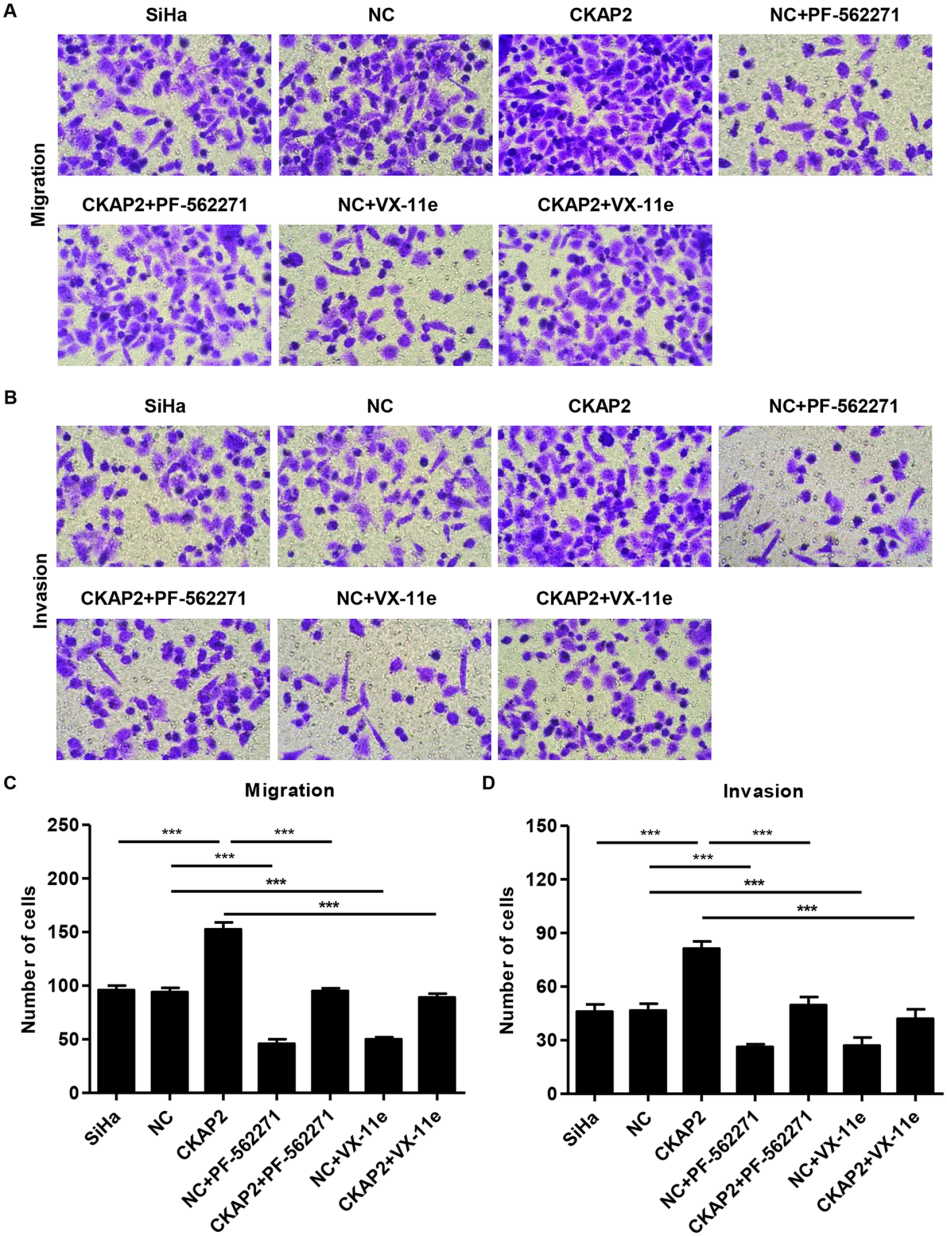
Effects of PF-562271 or VX-11e treatment on SiHa cell migration and invasion. (**A**,**C**) SiHa cells were treated with 10 μM of PF-562271 or VX-11e, and cell migration was measured by Transwell assay. (**B**,**D**) SiHa cells were treated with 10 μM of PF-562271 or VX-11e, and cell invasion was measured by Transwell Matrigel assay. ***P < 0.001.

Further exploration of the mechanisms involved in CKAP2 was done by examining the effect of CKAP2 overexpression on cell motility after infection with pLVX-Puro-CKAP2 or empty vector in the presence of FAK inhibitor PF-562271 or ERK2 inhibitor VX-11e in SiHa cells. The results of Real-time PCR and western blot analysis showed that the expression of CKAP2 was significantly upregualted in SiHa cells infected with pLVX-Puro-CKAP2 compared to those with empty vector (Fig. 6A), but PF-562271 (10 μM) treatment for 48 h significant decrease in CKAP2 expression levels (Fig. 6B). Moreover, PF-562271 (10 μM) or VX-11e (10 μM) treatment significantly decreased the expression level of p-ERK2, while CKAP2 overexpression increased the p-ERK2 level (Fig. 6C). No significant differences were observed in the expression levels of ERK2 in SiHa cells with different treatments. As shown in Fig. 7A–D, CKAP2 overexpression increased the cell migration and invasion of SiHa cells compared with controls. However, PF-562271 (10 μM) or VX-11e (10 μM) treatment significantly decreased cell migration and invasion induced by CKAP2 overexpression in SiHa cells. These data confirm that CKAP2 functions as an oncogene by FAK and ERK2 activation in cervical carcinoma.

## Discussion

The CKAP2 has been shown to be upregulated in many cancer tissues and cell lines, suggesting an important role of CKAP2 in the highly proliferative trait of cancers. Previous study showed that 50% of gastric adenocarcinomas observed CKAP2 expression, but no protein was detected in normal mucosal cells^10^. Furthermore, CKAP2 mRNA expression was greater in hepatocellular carcinoma than in nontumor tissues, whereas in some cases the CKAP2 mRNA expression appeared contrast to the protein level according to immunohistochemistry^12^. In the present study, we found that CKAP2 was upregulated in cervical carcinoma and HPV-16-positive CIN III tissues compared with either adjacent tissues or nontumor tissues both in mRNA and/or protein levels. The increased CKAP2 expression was significantly correlated with age, FIGO stage, lymph node metastasis, recurrence and tumor size. Persistent infection with HPV has been causally linked to the majority of cervical cancers and the role of HPV testing in cervical cancer screening is increasing, so much so that primary screening with only HPV testing is being considered as a new screening modality^21^. In the present study, 20 HPV genotypes were found in patients with cervical carcinoma, including HPV6/11/16/18/31/33/34/35/40/42/43/45/51/52/54/56/58/59/70/73 (Supplementary Table 1). However, CKAP2 expression was not associated with clinical characteristics, including HPV types, in our samples from hospital data. In addition, highly expressed CKAP2 observed both in HeLa and C-33A cells which carry positive and negative HPV, respectively. These results suggest that no directly correlation between CKAP2 expression and HPV types. Although the role of overexpressed CKAP2 in some human malignancies, including cervical carcinoma, as shown in this study, remains unclear, overexpression of CKAP2 has been shown to result in the development of monopolar spindles and subsequent arrest at prometaphase, leading to spindle defects^8^.

To further investigate the effect of CKAP2 on cervical carcinoma tumorgenesis, CKAP2-shRNA expressing vector was established and infected into cervical carcinoma cell lines. As we shown, knockdown of CKAP2 significant decreases in cervical carcinoma cell proliferation and tumor growth *in vivo*, which in line with other report that knockdown of CKAP2 reduced pRB phosphorylation and increased p27 expression, and consequently reduced human foreskin fibroblasts proliferation, whereas constitutive CKAP2 expression enhanced proliferation^7^, suggesting a critical role of CKAP2 in cell proliferation. As cancer cells lose the ability to stop at G1 and divide continuously, they always express CKAP2. Contrast to our findings, Tsuchihara *et al*.^14^ showed that CKAP2 transfection reduced colony formation and the proportion of colon cancer cells HCT116 that was in S phase, and induced aneuploidy leading to genomic instability and tumorigenesis rather than cell death. Overexpression of CKAP2 in prostate cancer cells C4-2B4 also decreased cell numbers and also involved in the RSK-mediated prostate cancer survival, consistent with the function of CKAP2 in cell-cycle inhibition^22, 23^.

MMP-2 and MMP-9, are potent gelatinases and have been correlated with the processes of tumor cell invasion and metastasis, play an important role in the various pathologies such as development and progression of cancer^24^. Overexpression of MMP-2 and MMP-9 has been observed in pre-cancer and cancer lesions of the cervical uterine^25^ and involved in the progression of cervical uterine cancer^26^. In the present study, we found that metastasis-related marker, MMP2, MMP-9 and Snail, were significantly decreased by CKAP2 knockdown, except E-cadherin. E-cadherin repressor Snail is associated with epithelial-mesenchymal transition in cervical cancer^27^. MiR-203 inhibited migration and invasiveness of prostate cancer cell lines through targeting CKAP2^28^. These data suggest that CKAP2 plays an important role in metastasis progression of cervical carcinoma.

A large body of evidence supports the role of FAK in regulation of cell proliferation, survival, and migration. Inhibition of FAK in MTLn3 cells results in decreased proliferation *in vitro* as well as decreased primary tumor growth *in vivo* ^29^. A high correlation between increased FAK expression and a tumor’s invasive and/or metastatic potential was not surprisingly found in other studies^30, 31^. Weak expression of FAK in patients with cervical cancer is specifically correlated with pelvic lymph node metastasis and recurrent disease, resulting in a poor disease outcome^32^. However, Oktay *et al*. reported that FAK overexpression was shown in cervical cancer and linked to invasion and migration^16^. Altogether, these studies suggest that FAK may possess alternative roles in different tumors and/or in different stages of tumor progression. In our results, FAK protein was upregulated in cervical carcinoma tissues compared with adjacent tissues (Supplementary Fig. 5). Moreover, FAK signaling was shown associated with the expression of CKAP2, as well as the activation of ERK2 induced by CKAP2 overexpression, which was inhibited by PF-562271 treatment, a FAK inhibitor. VX-11e, an ERK2 inhibitor, inhibited the ERK2 activation in cervical carcinoma cells with either CKAP2 overexpression or not. FAK acts as a linker of transmembrane receptors with the major growth regulatory pathways, including extracellular signal-related kinase (ERK) and c-Jun-N-terminal kinase (JNK)/mitogen-activated protein kinase (MAPK) pathways^33^. Downregulating MMP-2/9 via inhibiting the activation of ERK/MAPK signaling pathways inhibited migration and invasion of colorectal cancer^34^. Activation of ERK1/2 signaling pathway also promotes the proliferation and invasion of cervical cancer Cells^35^. In line with the previous studies, more importantly in our study, the migration and invasion ability of cervical carcinoma cells was increased after CKAP2 overexpression, but was inhibited by either PF-562271 or VX-11e treatment. These results indicate that activation of FAK-ERK2 signaling pathway is implicated in the effect of CKAP2 on proliferation, migration and invasion in cervical carcinoma cells.

In conclusion, the present study, for the first time, suggests that CKAP2 acts as a functional oncogene in cervical carcinoma cell line, and the upregulation of CKAP2 expression is closely associated with cervical carcinoma cell proliferation and motility through FAK-ERK2 signaling pathway. Thus, these results indicate that CKAP2 may become a novel promising candidate for therapy for cervical carcinoma.

## Electronic supplementary material

Supplementary information
