# Supplementary material for: Involvement of FAK-ERK2 signaling pathway in CKAP2-induced proliferation and motility in cervical carcinoma cell lines
Source: Sci Rep. 2017 May 18;7:2117. doi: 10.1038/s41598-017-01832-y (PMC5437009; doi:10.1038/s41598-017-01832-y)
Supplement: Supplementary file 1 — Supplementary information [file 41598_2017_1832_MOESM1_ESM.doc]

**Involvement of FAK-ERK2 signaling pathway in CKAP2-induced proliferation and motility in cervical carcinoma cell lines**

Qi-sang Guo^1,3^, Yu Song^1,3^, Shu-jun Gao^1,3^, Ke-qin Hua^#2,3^

**Supplementary Table 1.** HPV genotype distribution amongst HPV-DNA cervical carcinoma positive cases

| HPV type | Cervical carcinoma N=247 HPV positive | |
| --- | --- | --- |
|  | N | (%) |
| HPV6  HPV11 | 2 | 0.8 |
|  | 1 | 0.4 |
| HPV16 | 158 | 64.0 |
| HPV18 | 58 | 23.5 |
| HPV31 | 2 | 0.8 |
| HPV33 | 6 | 2.4 |
| HPV34 | 1 | 0.4 |
| HPV35 | 2 | 0.8 |
| HPV39 | 0 | 0.0 |
| HPV40 | 1 | 0.4 |
| HPV42 | 1 | 0.4 |
| HPV43 | 1 | 0.4 |
| HPV45 | 1 | 0.4 |
| HPV51 | 2 | 0.8 |
| HPV52 | 2 | 0.8 |
| HPV53 | 0 | 0.0 |
| HPV54 | 1 | 0.4 |
| HPV56 | 1 | 0.4 |
| HPV58 | 1 | 0.4 |
| HPV59 | 1 | 0.4 |
| HPV66 | 0 | 0.0 |
| HPV68 | 0 | 0.0 |
| HPV70 | 1 | 0.4 |
| HPV73 | 1 | 0.4 |
| HPV74 | 0 | 0.0 |
| HPV undetermined | 3 | 1.2 |


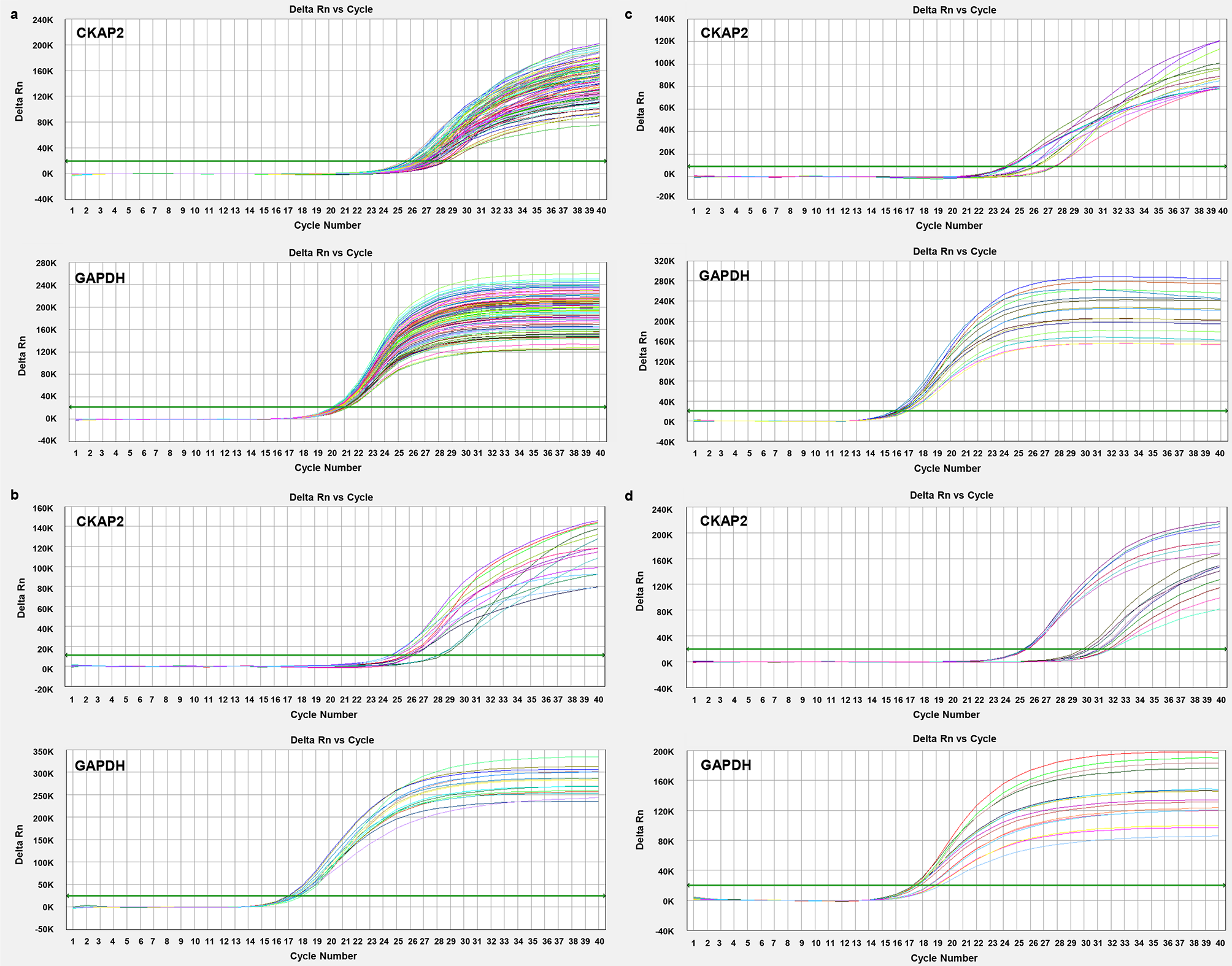


**Supplementary Figure 1. The Real-time PCR melting curves of CKAP2 and GAPDH.** Representative Real-time PCR melting curves of PLK2 and GAPDH in Figure 1C (a), 2A (b), 2C (c) and 2D (d) were shown.

**
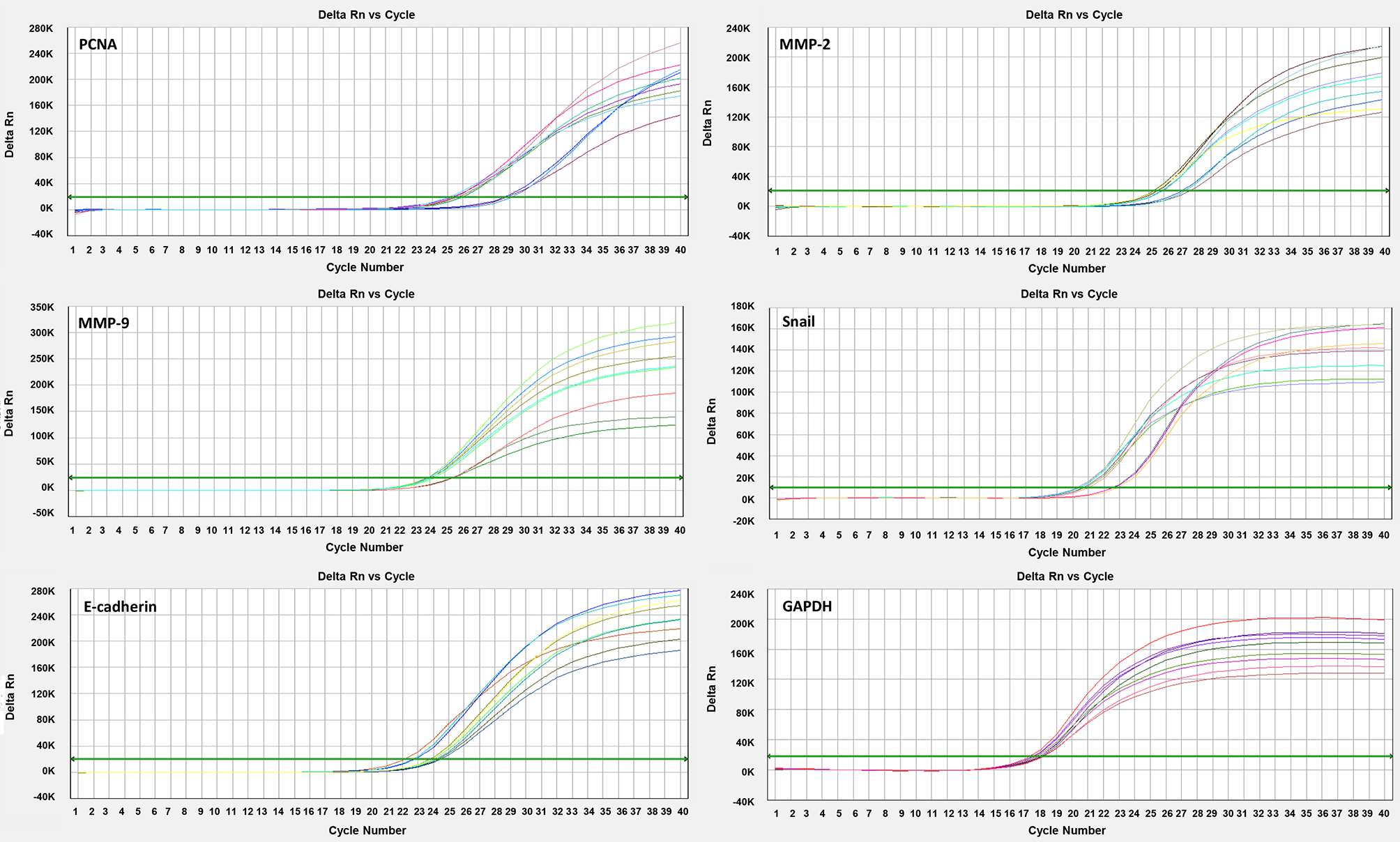
**

**Supplementary Figure 2. The Real-time PCR melting curves of PCNA, MMP-2, MMP-9, Snail, E-cadherin and GAPDH in Figure 5A.**

**
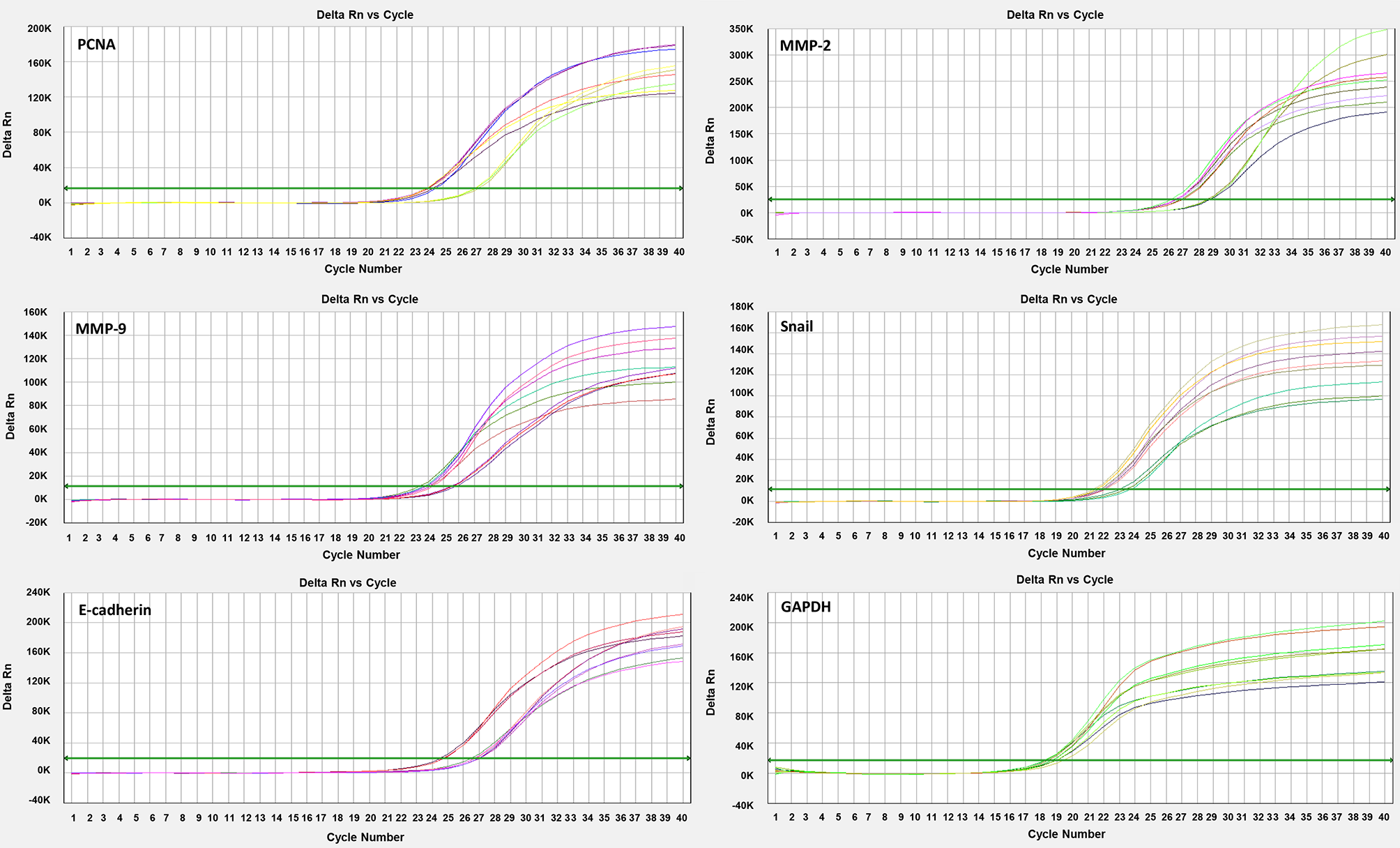
**

**Supplementary Figure 3. The Real-time PCR melting curves of PCNA, MMP-2, MMP-9, Snail, E-cadherin and GAPDH in Figure 5B.**

**
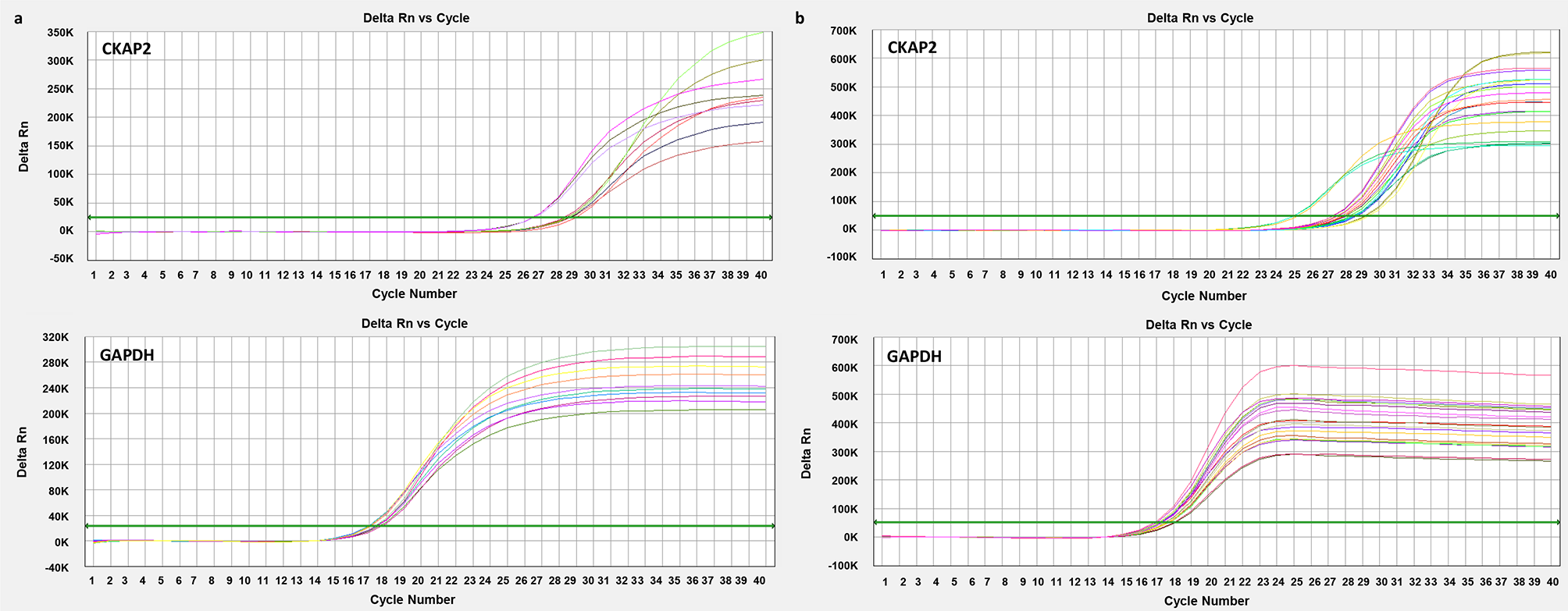
**

**Supplementary Figure 4. The Real-time PCR melting curves of CKAP2 and GAPDH.** Representative Real-time PCR melting curves of PLK2 and GAPDH in Figure 6A (a) and Figure 6B (b) were shown.


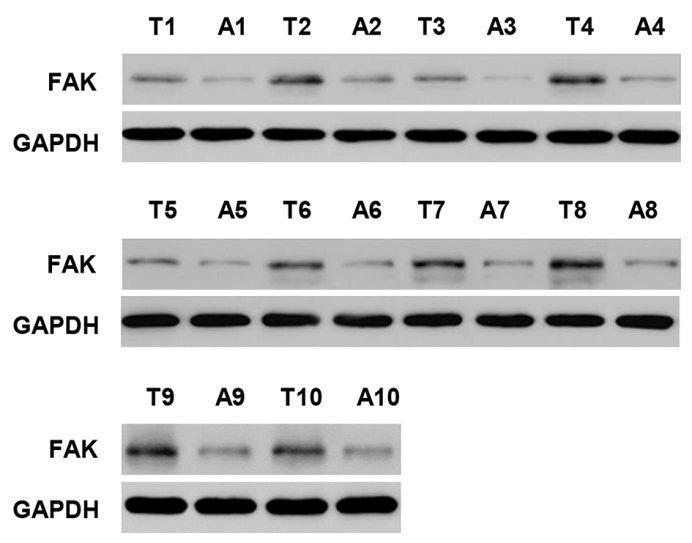


**Supplementary Figure 5. Expression of FAK in cervical carcinoma tissues.** Expression of FAK in ten primary cervical carcinoma tissues and their corresponding adjacent tissues measured by Western blot.
